# The Chagas disease study landscape: A systematic review of clinical and observational antiparasitic treatment studies to assess the potential for establishing an individual participant-level data platform

**DOI:** 10.1371/journal.pntd.0009697

**Published:** 2021-08-16

**Authors:** Brittany J. Maguire, Prabin Dahal, Sumayyah Rashan, Roland Ngu, Anca Boon, Colin Forsyth, Nathalie Strub-Wourgaft, Eric Chatelain, Fabiana Barreira, Sergio Sosa-Estani, Philippe J. Guérin

**Affiliations:** 1 Infectious Diseases Data Observatory (IDDO), Oxford, United Kingdom; 2 Centre for Tropical Medicine & Global Health, Nuffield Department of Medicine, University of Oxford, Oxford, United Kingdom; 3 Drugs for Neglected Diseases *initiative* (DND*i*), New York, New York, United States of America; 4 Drugs for Neglected Diseases *initiative* (DND*i*), Geneva, Switzerland; 5 Drugs for Neglected Diseases *initiative* (DND*i*), Rio de Janeiro, Brazil; 6 Epidemiology and Public Health Research Center, (CIESP-CONICET), Buenos Aires, Argentina; University of California San Diego, UNITED STATES

## Abstract

**Background:**

Chagas disease (CD), caused by the parasite *Trypanosoma cruzi*, affects ~6–7 million people worldwide. Significant limitations still exist in our understanding of CD. Harnessing individual participant data (IPD) from studies could support more in-depth analyses to address the many outstanding research questions. This systematic review aims to describe the characteristics and treatment practices of clinical studies in CD and assess the breadth and availability of research data for the potential establishment of a data-sharing platform.

**Methodology/Principal findings:**

This review includes prospective CD clinical studies published after 1997 with patients receiving a trypanocidal treatment. The following electronic databases and clinical trial registry platforms were searched: Cochrane Library, PubMed, Embase, LILACS, Scielo, Clintrials.gov, and WHO ICTRP.

Of the 11,966 unique citations screened, 109 (0.9%) studies (31 observational and 78 interventional) representing 23,116 patients were included. Diagnosis for patient enrolment required 1 positive test result in 5 (4.6%) studies (2 used molecular method, 1 used molecular and serology, 2 used serology and parasitological methods), 2 in 60 (55.0%), 3 in 14 (12.8%) and 4 or more in 4 (3.7%) studies. A description of treatment regimen was available for 19,199 (83.1%) patients, of whom 14,605 (76.1%) received an active treatment and 4,594 (23.9%) were assigned to a placebo/no-treatment. Of the 14,605 patients who received an active treatment, benznidazole was administered in 12,467 (85.4%), nifurtimox in 825 (5.6%), itraconazole in 284 (1.9%), allopurinol in 251 (1.7%) and other drugs in 286 (1.9%). Assessment of efficacy varied largely and was based primarily on biological outcome; parasitological efficacy relied on serology in 67/85 (78.8%) studies, molecular methods in 52/85 (61.2%), parasitological in 34/85 (40.0%), microscopy in 3/85 (3.5%) and immunohistochemistry in 1/85 (1.2%). The median time at which parasitological assessment was carried out was 79 days [interquartile range (IQR): 30–180] for the first assessment, 180 days [IQR: 60–500] for second, and 270 days [IQR: 18–545] for the third assessment.

**Conclusions/Significance:**

This review demonstrates the heterogeneity of clinical practice in CD treatment and in the conduct of clinical studies. The sheer volume of potential IPD identified demonstrates the potential for development of an IPD platform for CD and that such efforts would enable in-depth analyses to optimise the limited pharmacopoeia of CD and inform prospective data collection.

## Author summary

Chagas disease, also known as American trypanosomiasis, is a neglected tropical disease transmitted by triatomine insects, first identified in 1909. Chagas disease affects approximately 6–7 million people globally and is highly prevalent in Latin America where most cases are reported. However, there is increasing evidence that Chagas disease is now an important public health issue outside the “classical” endemic countries due to population migration. Our understanding of Chagas disease, including its pathologies and factors relating to progression, remains to date limited, and is also challenged by lack of diagnosis and highly effective treatment. This systematic review aims to describe studies with Chagas patients receiving antiparasitic treatment. Databases were searched for relevant studies published after 1997, and the results of these searches were screened.

Although a large volume of studies was identified in the review, heterogeneity was observed in study design, diagnostic methods, outcome assessment, and treatment regimens. While this aspect will be a limitation in pooling individual patient data, the volume of data available should allow sufficient comparison to form the basis of guidelines for future studies. The results of this review demonstrate that development of a Chagas disease data platform for clinical research would enable optimisation of existing data to strengthen evidence for the treatment and diagnosis of Chagas disease.

## Introduction

### Background

Chagas disease, caused by the protozoan parasite *Trypanosoma cruzi*, is considered a neglected tropical disease (NTD) [1]. Infection can occur via vectorial, congenital, oral or iatrogenic routes. Endemic in Latin America, with communities of affected people in non-endemic countries as well, Chagas disease has a high burden of morbidity and mortality affecting ~6–7 million people worldwide [1–4].The disease has two distinct phases; an acute phase that lasts up to two months and is usually asymptomatic, and the chronic phase that may last for decades [2]. The chronic phase may be either asymptomatic (or indeterminate) during which the patients can transmit the infection or symptomatic (develops among 10% to 40% of the patients) that can lead to cardiomyopathies and digestive tract pathologies. The efficacy of existing drugs has been well-studied in the chronic indeterminate phase; however, there are several questions that remain unanswered [2]. For example, it remains unclear if achievement of parasite clearance post treatment can prevent long term disease progression, the role of immunity in parasite persistence, risk factors predicting long-term progression observed in a subset of patients, biomarkers of parasite clearance or biomarkers of clinical cure, and optimal treatment regimens for the diversity of patient populations. A further challenge is an estimated lack of diagnosis and systematic treatment in more than 90% of patients globally [1,2]. Two nitro-heterocyclic drugs, benznidazole and nifurtimox, developed decades ago, are recommended for the treatment of the acute and early chronic phase of infection; however, availability of the drugs is limited and the treatments are hindered by poorly tolerated side effects. Furthermore, a lack of indicators to assess treatment efficacy impacts the use of these therapies in the different phases of the disease [2,5–7]. Antitrypanosomal treatment has been shown to prevent congenital transmission when women are treated prior to pregnancy and, according to several observational studies, may limit the development of complications from Chagas disease [8–10]. These challenges highlight an urgent need for improved access to currently available treatments in the short term but also a clear need for efficacious and safer drugs as well as biomarkers of cure for the future [2].

Existing data, collected in past clinical trials and longitudinal observational studies, can be used to generate new evidence, to address several knowledge gaps and guide research priorities. This approach is of great value in the context of neglected diseases, as seen with Chagas where there is a severe under-diagnosis of affected people; many studies are conducted in small, isolated samples, and there is a lack of large-scale research with sufficient power and robustness to fully answer some key questions about the disease. However, heterogeneity in the design and reporting of published studies and wide-ranging efficacy estimates in Chagas trials limit the usefulness of traditional aggregated meta-analyses [11–13]. These limitations could be circumvented by access to individual participant data (IPD) which could be standardised to a common standard and pooled to support more in-depth analyses and comparisons across individuals, regimens, or subgroups of interest as well as further defining knowledge gaps which could be addressed in future studies [14–16].

Chagas disease has a unique challenge in that the only reliable test to assess cure is seroconversion, which can take years following treatment and varies from person to person. Given current limits in our understanding of disease progression and natural history, and the need to continually monitor this chronic disease, there are calls to establish a maintained patient registry for Chagas disease as one tool to address critical research questions [17]. Meanwhile, retrospective data from clinical studies with long-term follow-up could be utilised immediately, provided that we assemble the data rapidly, acknowledging that research data are lost steadily over time, often due to an inability to contact authors and obsolete data storage systems [18]. Therefore, it is important to promptly identify a comprehensive and reliable platform to gather this important information.

Success of such an initiative in the context of malaria has been demonstrated by the WorldWide Antimalarial Resistance Network (WWARN), a platform hosted by the Infectious Diseases Data Observatory (IDDO). This unique, decade-long collaborative data sharing framework has shown it is possible to produce policy-changing scientific evidence from historical data [19–23] and address questions of public health relevance [24–29]. IDDO has now expanded beyond malaria clinical trial data to facilitate data platforms for other neglected diseases and emerging infections including visceral leishmaniasis, schistosomiasis and soil-transmitted helminthiases, Ebola, and COVID-19.

The Drugs for Neglected Diseases *initiative* (DND*i*) has been leading the development of drug regimens for many NTDs including Chagas disease. DND*i* has asked IDDO to conduct a systematic review of clinical studies in Chagas disease to gauge the potential of establishing a data repository to facilitate further drug development.

### Rationale & objectives

It was deemed that a similar approach to the collation, harmonisation, access and re-use of IPD from Chagas clinical studies would be an important tool permitting secondary analyses which could lead to valuable new insights, enhance the reproducibility and credibility of prospective clinical research, and honour the contributions of trial participants. A project was initiated by the DND*i*, which aligns with calls for greater transparency and data sharing in clinical research that are gaining traction globally [21,30–35]. Data sharing and open science is an asset for researchers and innovation, and is also good for patients because it maximises the scientific application of existing data, valuing each participant’s contribution and allowing more efficient research-driven responses to improve patient care [36].

As part of an initial phase to determine the potential of establishing such a platform for the Chagas research community, a systematic literature review was conducted with three primary objectives. First, to understand the scope of existing Chagas clinical studies and availability of data. Second, to characterise Chagas trypanocidal treatment efficacy studies conducted in the last 20 years. From this characterisation, including investigation of heterogeneity in study design and methodologies, the final objective was to assess the technical value and feasibility of establishing an IPD platform. Here, we present the results of this systematic review.

## Methods

The protocol for this systematic review was prospectively registered on PROSPERO CRD42017076939. This review was conducted in accordance with the Preferred Reporting Items for Systematic-Reviews and Meta-Analyses (PRISMA) guidelines (S1 Text).

### Literature searches

The search strategy was developed in collaboration with an experienced librarian (Nia Roberts). The following electronic databases and clinical trial registry platforms were searched: Cochrane Library, PubMed, Embase, LILACS, Scielo, Clintrials.gov and WHO ICTRP. The searches were restricted to January 1997 onwards as a method of identifying all studies that were completed between January 1997 and the date of final searches (23 August 2019), due to the significantly reduced likelihood of data retrieval with longer time since publication [18]. We used the search terms “*trypanosoma cruzi*”, “chagas disease”, “chagas” OR “American trypanosomiasis”, and where possible applied filters to limit results to publications with a human population. Filters to exclude secondary sources (e.g. letters and editorials) were also implemented in some databases. We did not place any restrictions on the language of publication. Articles in non-English were assessed using google translation. A comprehensive list of search terms by database is available in supplemental file (S2 Text).

### Screening and eligibility criteria

After duplicates were removed, the title and abstracts from the searches were screened by a single reviewer [BM] using Covidence software for systematic reviews [37]. Although not blinded, all citations that were screened at the level of full-text review for inclusion were assessed by at least two reviewers [BM, AB, SR]. Eligibility was assessed against pre-determined inclusion and exclusion criteria, described elsewhere (S2 Text). To summarise, all prospective clinical studies completed after January 1997 enrolling patients with a confirmed Chagas diagnosis, exposure to a trypanocidal treatment and at least one follow-up measurement were eligible for inclusion in the analysis. During screening, all prospective clinical studies that did not expose Chagas patients to a trypanocidal treatment, such as longitudinal observational studies with no treatment exposure, as well as studies assessing symptomatic interventions for Chagas sequelae, were flagged to understand the scope of Chagas clinical research, before their exclusion from further data extraction and investigation of trial characteristics.

Studies with immunosuppressed patients were included and no limits were applied to participant age or Chagas disease phase. Further, publications were not excluded by outcome given the desire to compare and to understand the homogeneity/heterogeneity of all outcomes measured in Chagas disease. Case studies, studies with less than 6 Chagas disease patients, non-primary publications, cross-sectional studies, and epidemiological studies were all excluded. Additionally, we excluded all studies with no post-treatment follow-up. Reviews and meta-analyses obtained from the search were subsequently hand-searched for potentially relevant results.

### Data extraction

Data were extracted from all eligible studies using a standardised variable dictionary (S1 Data) [in a pre-piloted REDCap[38,39] database designed to collect information on study location, design, population, intervention, follow-up and outcome measures. Data items for extraction were modified and extended from a previous systematic review by Julé et al (2016) [40]. All studies were initially extracted by one of the reviewers [AB, SR or RN] and then cross-checked by another. Any discrepancies were resolved by discussion or a third reviewer [BM].

### Synthesis of results

Descriptive statistics were used to present the extracted data. Categorical variables were summarised with proportions and frequencies; continuous variables were summarised with totals, means, quartiles, minimums and maximums. Summary statistics and figures were produced using R software (version 3.6.3, the R Foundation for Statistical Computing, Vienna, Austria). For the purposes of this review, we use the term “study arm” to refer both to a study arm from a clinical trial and a patient cohort from an observational study where treatment is administered.

### Risk of bias

Although stipulated in the protocol, owing to the volume of studies identified and primary objectives, meta-analysis of study results was not conducted and this review does not address specific clinical questions. Results are limited to the initial data extraction, descriptive analysis of trial characteristics, study design and interventions. Accordingly, assessment of meta-biases or the strength of the body of evidence represented within and across individual included records are not relevant. However, the biases related to the identification and inclusion of literature in this review and totality of evidence presented are qualitatively discussed and assessed using the GRADE approach [41].

## Results

### Search results

**Fig 1:**
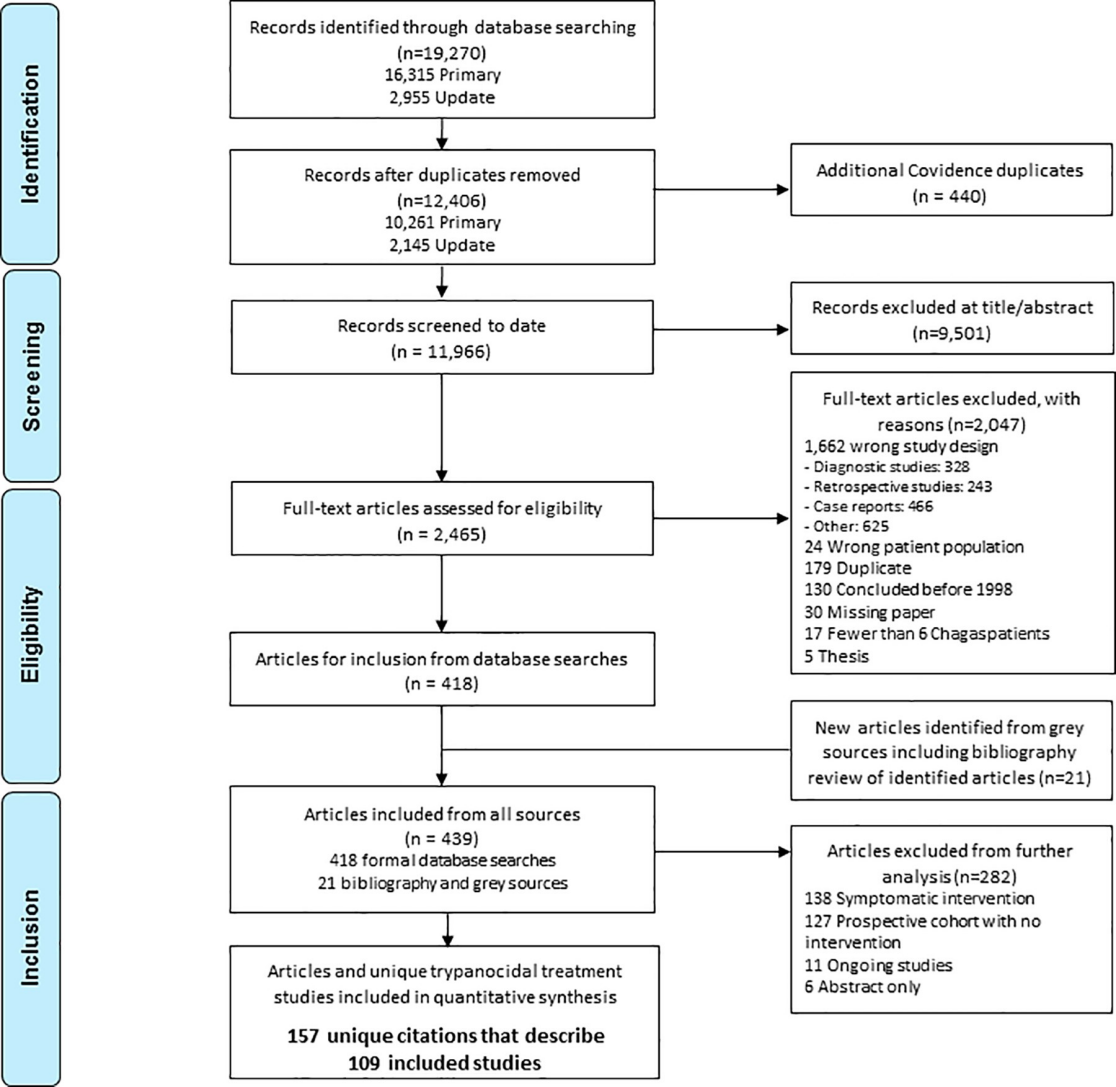
Preferred Reporting Items for Systematic Reviews and Meta-Analyses (PRISMA) flow diagram of publications screened.

The identification of studies included in this review is the result of primary literature searches conducted on 7-11^th^ September 2017, with update searches of all databases subsequently conducted on 23^rd^ August 2019. A PRISMA flowchart presented in Fig 1 provides a graphic depiction of the results of the searches. Across both primary and update searches (n = 19,270), after the removal of duplicates (n = 7,304), a total of 11,966 unique citations were identified. A total of 9,501 citations were excluded at the level of title and abstract screening and 2,465 full-text articles were assessed for eligibility against pre-determined inclusion criteria (S2 Text). Among the 2,047 publications excluded at the full-text level, 328 described diagnostic accuracy studies without treatment assessment, 243 were retrospective Chagas studies and 466 were case reports. A total of 418 publications met the full-text inclusion criteria for screening. An additional 21 articles were identified from bibliography review of included publications and grey sources. Due to the volume of identified literature, and to understand the scope of Chagas clinical research, prospective studies assessing symptomatic interventions (n = 138) or observational cohorts where no intervention was routinely administered and assessed in all included participants (n = 127) were categorised before exclusion from further quantitative analysis. The remaining 157 citations, which describe 109 unique clinical studies assessing a trypanocidal treatment with follow-up, met the pre-defined inclusion criteria for quantitative assessment. Of the 109 studies, 87 (80%) were published in English language, 10 (9%) in Portuguese and 12 (11%) were in Spanish. Results of the literature searches and included articles can be found in supplemental files (S2 Data, S3 Data).

### Analysis of trypanocidal treatment study characteristics

Of the trypanocidal treatment studies included for further data extraction, a total of 109 studies published between 1997 and the end of 2019 were included in the quantitative synthesis of this review. Results of data items collected about information pertaining to study location, design, population, intervention, follow-up and outcome measures are presented herein.

### Spatial and temporal distribution

**Fig 2:**
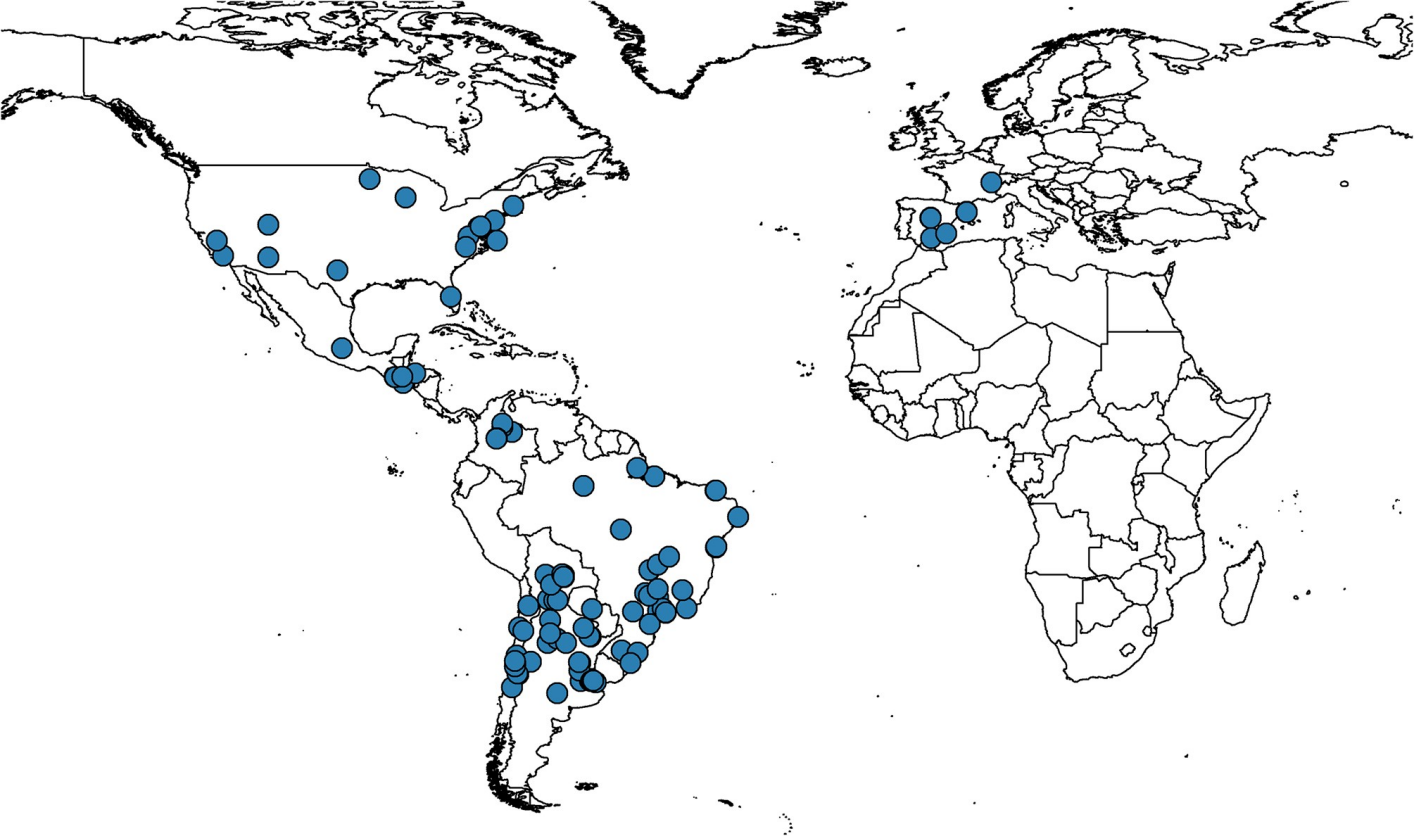
Sites where the studies included were conducted.

The large majority of the studies were conducted in the Americas while some were conducted more recently in Spain and Switzerland (Fig 2). Seventy-seven (70.6%) studies were from the Americas, 24 (22.0%) were from Europe, 3 (2.8%) were multi-regional and the region of data origin was not clear in 5 (4.8%). A total of 6 (5.5%) studies were published prior to 2000, 31 (28.4%) published from 2000 to 2009 inclusive and 72 (66.1%) from 2010 to 2019. From the 109 studies, there was a total of 161 cohorts from 14 countries. There were 50 (31.1%) cohorts from Argentina, 32 (19.9%) from Brazil, 31 (19.3%) from Spain, 13 (8.1%) from Bolivia, 13 (8.1%) from Chile and a breakdown for the remaining 22 (13.6%) cohorts is presented in Fig 2. A total of 47 (29.2%) study cohorts were from urban areas, 12 (7.5%) from rural areas, 1 (0.6%) from semi-urban areas, 8 (4.9%) cohorts were from a mixture of urban or rural settings, and the location was not clear in the remaining 93 (57.8%) cohorts.

**Fig 3:**
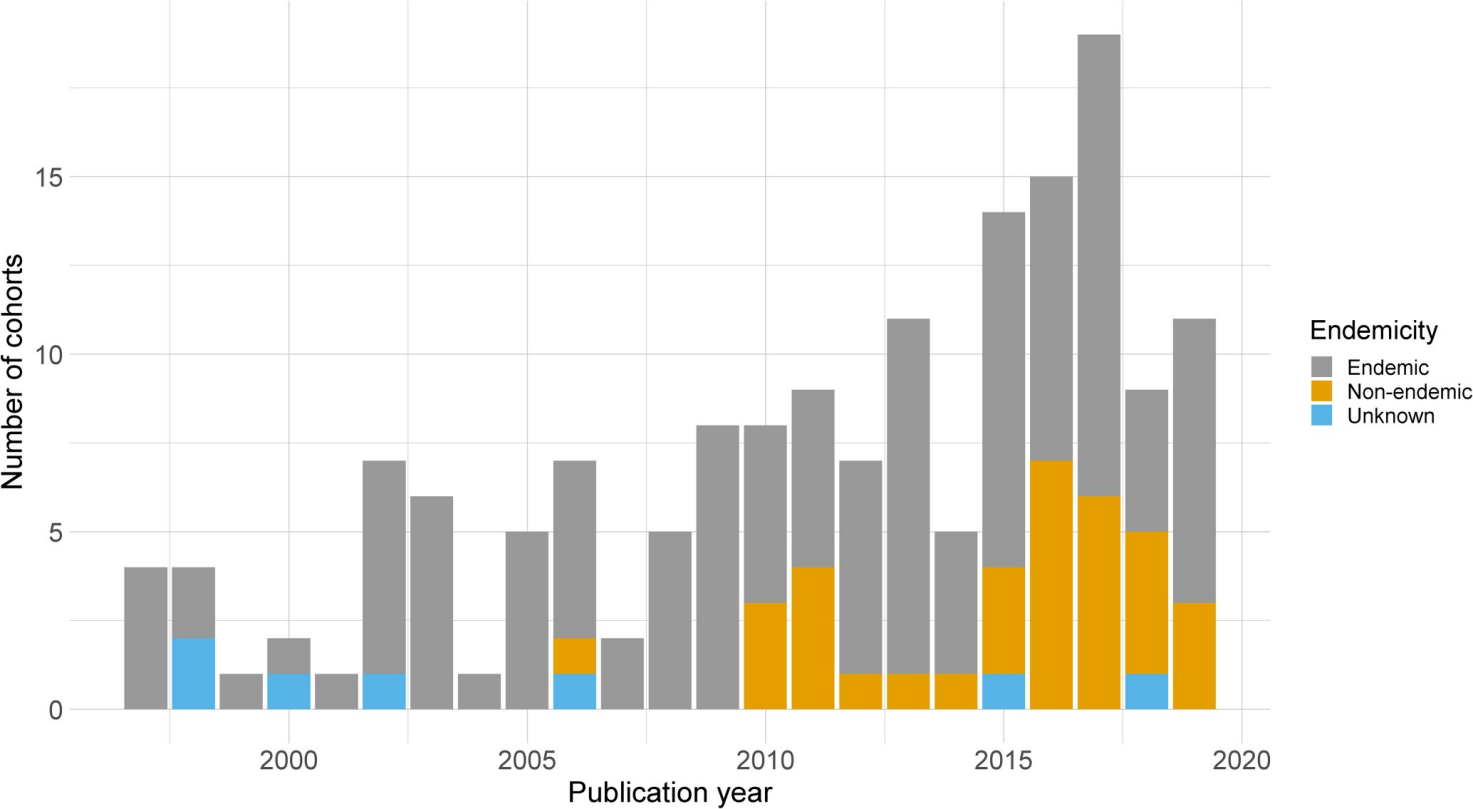
Temporal distribution of cohort endemicity.

Disease endemicity status was reported for 154 (95.6%, 154/161) study cohorts (endemicity assigned as per WHO [42], see Variable dictionary, S1 Data) of which 120 (77.9%) were from endemic countries and 34 (22.1%) were from non-endemic countries (Fig 3). Prior to 2010, only 1 (1.9%, 1/53) of the study cohorts was conducted in a non-endemic region whereas 33 (30.6%, 33/108) of the study cohorts in studies published from 2010 to 2019 were from non-endemic regions (Fig 3). The median sample size of participants enrolled per cohort was relatively small with 45 patients [Interquartile range (IQR): 13–103; range: 1–3,579].

### Study design

Thirty-three (30.3%) studies were observational (31 were cohort studies, 1 was a diagnostic accuracy study assessing treatment, and 1 was a case series) and 76 (69.7%) were interventional. Of the latter, 63 (82.9%, 63/76) were non-randomised, 11 (14.5%) were randomised, and 2 (2.6%) were quasi-randomised. The duration of follow-up was less than 1 year in 32 (29.4%) trials, between 1 to 5 years in 50 (45.9%), 5 to 10 years in 15 (13.8%), 10 to 15 years in 5 (4.6%) and greater than 15 years in 7 (6.4%) (See S3 Data for further study meta-data).

### Study population

**Fig 4:**
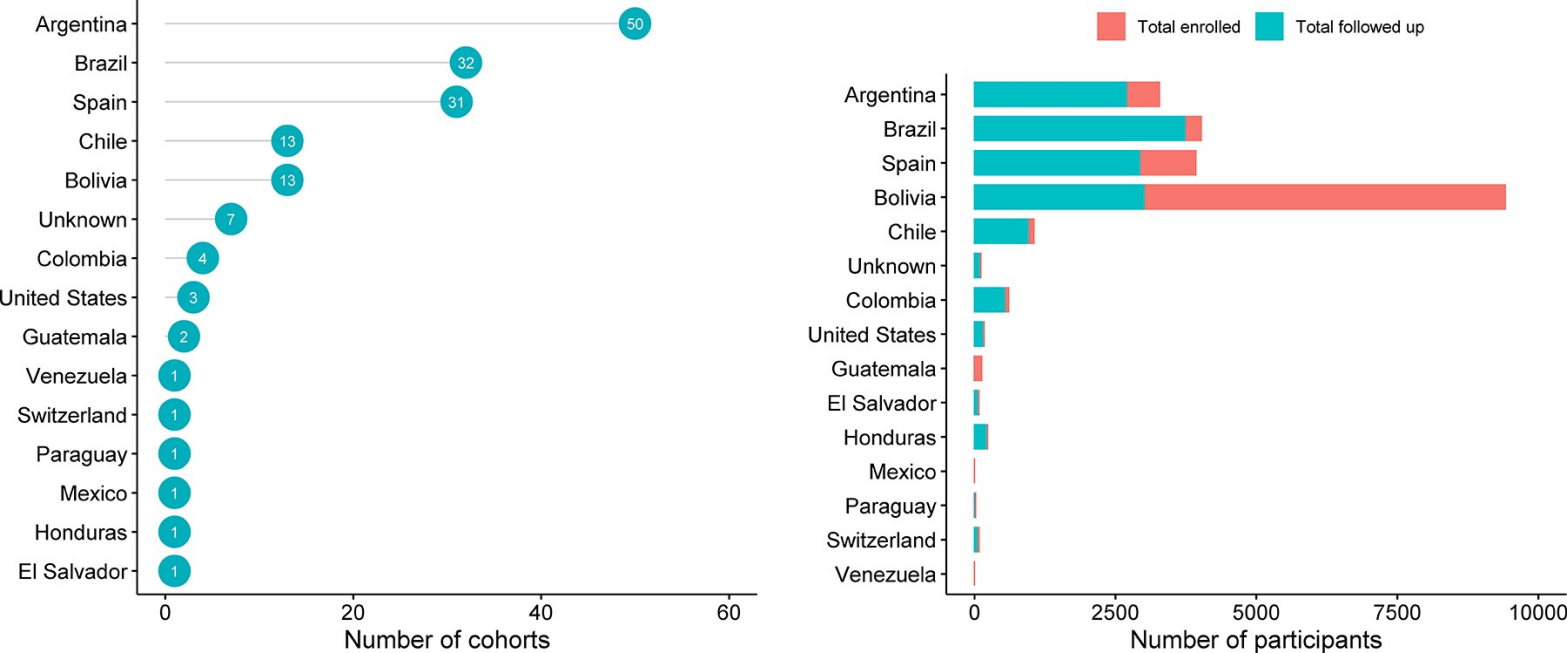
Number of cohorts and participants size stratified by country.

From 161 study cohorts included in this review, there were a total of 25,827 participants enrolled of which 23,116 (89.5%) were patients. The top country of origin for patients was Bolivia (9,415, 40.7%) followed by Brazil (4,024, 17.4%), Spain (3,924, 17.0%), Argentina (3,280, 14.2%) and Chile (1,054, 4.6%); the remaining participant distribution is presented in Fig 4. Of the 3,924 patients from Spain, 3220 (82.1%) were of Bolivian origin, 16 (0.4%) were infants with congenital disease, and majority of the remaining 540 (13.8%) were also of Bolivian origin (the exact number of patients couldn’t be clearly ascertained). A breakdown on the number of participants enrolled by gender was reported in only 64 (58.7%) studies that enrolled a total of 11,958 patients (5,196 males and 6,762 females). Participants aged <2 years were included in 3 (2.8%) studies, participants aged 3 to 12 years in 6 (5.5%) studies, participants aged 13 to 17 years in 1 (0.9%) study, participants aged 18+ years in 51 (46.8%) studies, 44 (40.4%) studies included participants from two or more of the preceding age groups, and the age distribution was unclear in 4 (3.7%) studies. Pregnant women were included in 5 (4.6%) studies and excluded in 48 (44.0%); this information was not clear in the remaining 56 (51.4%) studies. Malnourished patients were excluded in 5 studies (4.6%) and this information was not clear in the remaining 104 (95.4%). Immunocompromised patients were included in 5 (4.6%) studies and excluded in 20 (18.3%), while this information was unclear in 84 (77.1%). Six (5.5%) studies included patients in the acute phase of the disease, 86 (78.9%) in the chronic phase, 8 (7.3%) trials enrolled patients in either chronic or acute phase, and the phase of the disease was not reported in 9 (8.3%) (See S3 Data for further details).

### Diagnostic criteria used for patient enrolment

Fifty-five (50.5%) studies used serological methods for confirmation of the disease, 2 (1.8%) trials used molecular methods, 2 (1.8%) used parasitological methods, 42 (38.5%) trials used one or more combinations of serological, parasitological or molecular methods for confirmation of the disease and the diagnostic method used was not clear in 8 (7.3%) trials (Table 1). Five (4.6%) trials required only 1 positive test for disease confirmation (2 studies used polymerase chain reaction (PCR) methods, 1 study used PCR and serology, 2 studies used serology and parasitological methods), 60 (55.0%) trials required 2 positive tests, 14 (12.8%) required 3 positive tests, 4 (3.7%) required 4 or more positive test results and this information was not reported in 26 (23.9%) (Table 1). In the 6 studies in the acute phase of the disease, a parasitological method was used in 1 (16.7%), a molecular method in 1 (16.7%) and a combination of serological and parasitological methods in 4 (66.7%). In the 86 studies that enrolled patients in the chronic phase of the disease, serological methods were used in 48 (55.8%), molecular in 1 (1.2%), parasitological in 1 (1.2%), a combination of serological/molecular or parasitological in 31 (36.0%), and the diagnostic method was not clear in the remaining 5 (5.8%) (See S1 Table).

**Table 1:** Diagnostic criteria used for patient enrolment.

	Serology	Parasitology	PCR	Combination of Serology/parasitology /PCR	Not specified	Overall
Number of studies	55	2	2	42	8	109
Number of positive test results required						
1	0 (0%)	0 (0%)	2 (100%)	3 (7.1%)	0 (0%)	5 (4.6%)
2	38 (69.1%)	0 (0%)	0 (0%)	22 (52.4%)	0 (0%)	60 (55.0%)
3	8 (14.5%)	0 (0%)	0 (0%)	6 (14.3%)	0 (0%)	14 (12.8%)
4	0 (0%)	0 (0%)	0 (0%)	3 (7.1%)	0 (0%)	3 (2.8%)
>4	0 (0%)	0 (0%)	0 (0%)	1 (2.4%)	0 (0%)	1 (0.9%)
Not specified	9 (16.4%)	2 (100%)	0 (0%)	7 (16.7%)	8 (100%)	26 (23.9%)

Percentages are based on columns; PCR = polymerase chain reaction

### Treatment regimen

**Fig 5:**
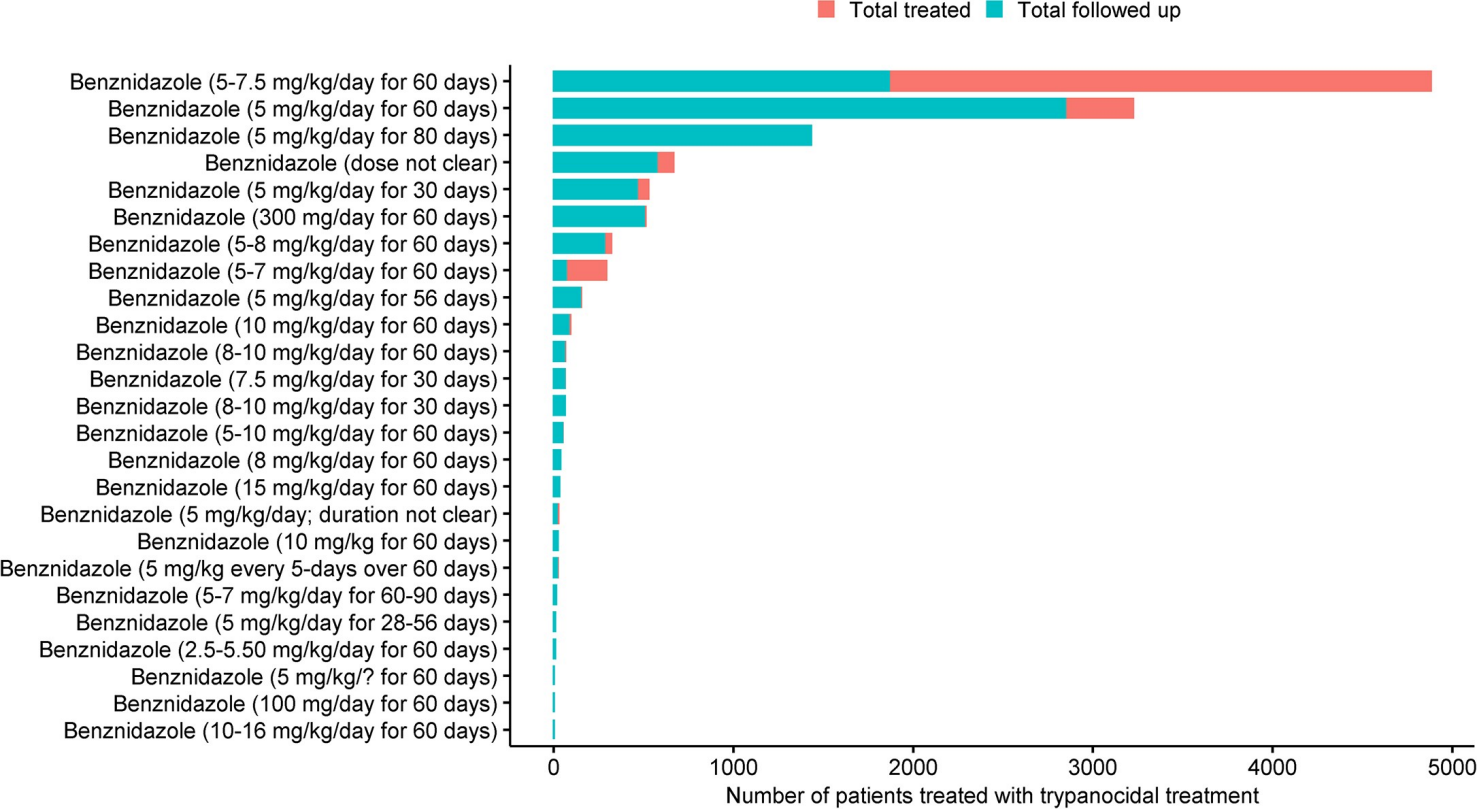
Body weight adjusted (mg/kg) drug dosage variability for benznidazole.

**Fig 6:**
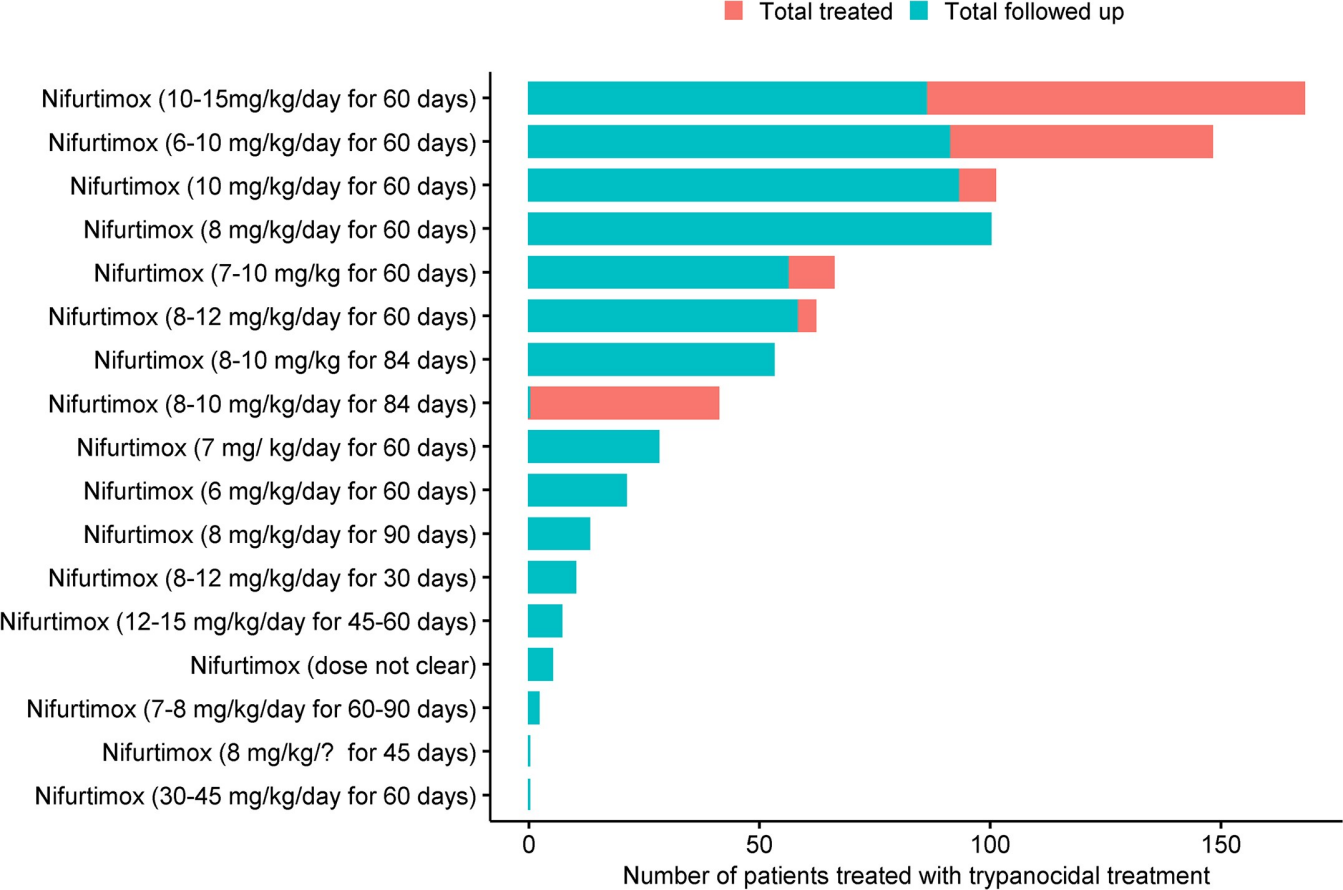
Body weight adjusted (mg/kg) drug dosage variability for nifurtimox.

Of the 23,116 patients who were enrolled, information on treatment status was available for 19,199 (83.1%) patients. Of these, 14,605 (76.1%) patients received an active treatment and 4,594 (23.9%) were actively assigned to a placebo or no treatment (Table 2). Benznidazole (as a monotherapy) was administered in 89 (51.1%) arms (12,467 patients) and in combination with other regimens in 14 (8.0%) arms (482 patients). There was a wide variability in the daily mg/kg/day drug dosage of benznidazole administered, with 5–7.5 mg/kg/day for 60 days tested in 2 arms (4,879 patients) being the most tested regimen followed by 5 mg/kg/day for 60 days (36 arms; 3,222 patients) (Fig 5). Among adults treated with benznidazole, 5 mg/kg/day for 60 days was the most tested regimen (2,299 patients; 42 study arms) followed by 5 mg/kg/day for 80 days (1431 patients, 1 arm). Among children, 5 mg/kg/day for 60 days was tested in 118 patients (2 arms), 5–10 mg/kg/day for 30 days in 121 patients (2 arms), and 5–10 mg/kg/day for 60 days in 145 patients (5 arms). In the remaining 44 arms, either patients of all age-ranges were included or the dosage was not clear (see S2 Table). Nifurtimox was administered in 19 (10.9%) arms (825 patients). Nifurtimox at a dosage of 10-15mg/kg/day for 60 days in 168 patients, 6–10 mg/kg/day for 60 days was tested in 148 patients, 10 mg/kg/day for 60 days in 101 patients, 8 mg/kg/day for 60 days in 100 patients, and the remaining breakdown in presented in Fig 6.

**Table 2:** Drug regimen tested in the studies included in the review.

Regimen	Number of study arms	Total number of patients treated	Total number of treated patients who were followed-up
Allopurinol	4 (2.3%)	251 (1.3%)	245 (1.7%)
Benznidazole	89 (51.1%)	12,467 (64.9%)	8,604 (59.2%)
Benznidazole + nifurtimox	2 (1.1%)	93 (0.5%)	93 (0.6%)
Benznidazole + other ^a^	12 (6.9%)	389 (2.0%)	383 (2.6%)
Itraconazole	4 (2.3%)	284 (1.5%)	222 (1.5%)
Nifurtimox	19 (10.9%)	825 (4.3%)	623 (4.3%)
Nifurtimox + other^a^	1 (0.6%)	10 (0.1%)	10 (0.1%)
No treatment/placebo	33 (19.0%)	4,594 (23.9%)	4,104 (28.2%)
Other^a^	4 (2.3%)	151 (0.8%)	150 (1.0%)
Posaconazole	3 (1.7%)	82 (0.4%)	75 (0.5%)
Unclear drug name	3 (1.7%)	53 (0.3%)	27 (0.2%)
Total	174	19,199	14,536

Percentages are based on columns

^a^other regimen includes Vitamins E and C, methylprednisolone, receiving allogenic bone marrow transplantation, thioctic acid, corticosteroids

### Assessment of parasitological outcome measures

Assessment of parasitological outcome measures was carried out in 85 (77.9%) studies of which negativisation of parasitaemia was assessed in 55 (64.7%); decrease in titre load, fluorescence activity, normalisation of hypercoagulability/changes in parasitaemia profile was assessed in 20 (24.0%) studies, and the parasitological outcome measures used was not clear in 10 (12.0%). Of the 55 studies that assessed negativisation, PCR was used in 13, serology in 20, parasitological methods in 5, culture in 1, and a combination of one or more method was used in 16 studies (Table 3). Only one method was used for assessment of antiparasitic effect in 27 studies (14 trials used only serological methods, 10 trials only used molecular and 3 used only parasitological), whereas the remaining 58 studies used a combination of different methods (Table 3). Overall, serological methods were used in a total of 67 (78.8%, 67/85) studies, molecular methods in 52 (61.2%), parasitological (including xenodiagnosis) in 34 (40.0%), immunohistochemistry in 1 (1.2%) and, microscopy in 3 (3.5%). Of the 52 studies that used molecular (PCR) methods, quantitative PCR was used in 18 (34.6%), qualitative PCR in 19 (36.5%), both quantitative and qualitative methods in 6 (11.5%), and an unspecified method in the remaining 9 (17.1%). During the study follow-up, there were 1 to 5 parasitological measurements in 44 (51.8%, 44/85) studies, 6 to 10 in 16 (18.8%) studies, 11 to 15 in 6 (7.1%) studies, 16 or more in 8 (9.4%), and the total number of measurements was not clear in the remaining 11 (12.9%) studies (S3 Table). The median time at which parasitological assessment was carried out was 79 days [IQR: 30–180 days] for the first assessment, 180 days [IQR: 60–500 days] for the second assessment and 270 days [IQR: 18–545 days] for the third assessment (See S4 Table).

**Table 3:** Parasitological outcome measures adopted and lab method used for measuring parasitaemia related endpoints.

	Number of studies (n = 85 studies)
**Parasitological measures adopted**	
Negativisation	
Negativisation of culture	1 (1.2%)
Negativisation using parasitology	5 (5.9%)
Negativisation using PCR	13 (15.3%)
Negativisation of serology	20 (23.5%)
Combination of one or more of the above	16 (18.8%)
Other measures	
Percentage of fluorescence positivity	1 (1.2%)
Decrease in titres /drop in reactivity compared to baseline or earlier time point/seroconversion	14 (16.5%)
Normalisation of hypercoagulability biomarkers	1 (1.2%)
Change in parasitaemia (or immune) profile	4 (4.7%)
Unclear	10 (11.8%)
**Lab method used for measuring parasitaemia related endpoints**	
Serological	14 (16.5%)
Molecular	10 (11.8%)
Parasitological	3 (3.5%)
Serological + molecular ^a^	27 (31.8%)
Serological + parasitological	14 (16.5%)
Molecular + parasitological	4 (4.7%)
Serological + molecular + parasitological	10 (11.8%)
Serological + parasitological + microscopy	1 (1.2%)
Parasitological + microscopy	1 (1.2%)
Serological + molecular + parasitological + immunohistochemistry + microscopy	1 (1.2%)

^a^ Molecular methods include polymerase chain reaction (PCR); Xenodiagnosis is considered as parasitological

### Assessment of patient related outcome measures

Patient related outcomes (PROs) were assessed in 96 (88.1%) studies. Of the 96 studies, adverse events were assessed in 75 (78.1%), clinical examination in 70 (72.9%) studies, laboratory or haematological assessment in 63 (65.6%), ECG in 31 (32.3%), immune response in 21 (21.9%) pharmacokinetics in 11 (11.5%) and the other PROs are presented in Table 4. Of the 96 trials that reported PROs, 6 (6.3%) described only 1 PRO, 15(15.6%) described two PROs, 25 (26.0%) described 3 PROs and 49 (51.0%) described 4 or more PROs.

**Table 4:** Patient related outcome measures.

Patient related outcomes	Number of studies ^a^	Number of patients enrolled	Number of patients followed-up
Adverse events	75 (68.8%)	19,218	11,195
Clinical examination	70 (64.2%)	15,802	7,941
Laboratory or haematological assessment	63 (57.8%)	7,798	7,079
Other outcome measures ^b^	38 (34.9%)	14,638	7,450
Electrocardiograph or ECG	31 (28.4%)	9,807	8,994
Immune response	21 (19.3%)	5,578	1,664
Disease progression	20 (18.3%)	8,392	4,558
Mortality	16 (14.7%)	13,777	6,512
Echocardiogram	14 (12.8%)	1,298	1,056
Pharmacokinetics	11 (10.1%)	729	703
**Overall**	**96 (88.1%)**	**22,271**	**14,124**

^a^ Percentage is out of the total number of trials (n = 109) included in the systematic review

^b^ Other patient related outcome measures assessed included (but not limited to) neurological examination, liver tests, heart disease progression, cardiologic evaluation, chest radiography, weight loss, treatment adherence, vertical transmission etc.

## Discussion

This systematic review identified 109 Chagas clinical studies enrolling 161 patient cohorts between 1997 and 2019, with nearly two-thirds of the studies published on or after 2010. A fifth of the study cohorts were from non-endemic regions (United States, Spain and Switzerland) with all but one of the cohorts coming from studies conducted in or after 2010. The increasing number of patient cohorts from non-endemic countries and increased volume of publications in the past 10 years indicates the recognition among scientists and funding agencies of the global distribution and burden of Chagas disease and the phenomenon of international migration. However, even with this substantial increase, the total volume of studies and evidence assembled over the last two decades remains limited and illustrates that Chagas disease is still neglected.

Given the volume of potential individual patient data identified in the analysis of this systematic review for trypanocidal treatment (n = 19,199) and the number of drug regimens/formulations tested, the potential of this individual patient data, were it to be standardised and pooled, is interesting. The most common trypanocidal treatment evaluated in included studies was benznidazole (treated patients, n = 12,467) followed by nifurtimox with only 825 treated patients. The dose range and regimen of administration for these two drugs across studies was vastly heterogeneous, as seen in Figs 5 and 6. This review also highlights the large heterogeneity of study design, diagnostic methods, and duration of follow-up and timing of outcome assessment used by investigators even in the last decade. While this aspect will be a limitation when pooling data, the volume of data available should allow identification of gaps to inform future research. Questions about the optimal regimen of the current drugs (benznidazole and nifurtimox) in chronic indeterminate patients, whether parasite clearance prevents progression of the disease, about the geographically patterned differences in *T*. *cruzi* and/or patient immune response, whether duration of infection before treatment has an impact on treatment outcome or if geographical location contributes to different treatment outcomes [16], cannot be answered with an aggregated meta-analysis but could be explored if individual patient data were made available [16,43].

As observed for most NTDs, pregnant women, patients with co-morbidities such as malnutrition, and immunocompromised patients are very often excluded from studies. While these populations would require specific therapeutic approaches, we have very limited evidence to guide the recommendations with, for example, only 5 studies that enrolled pregnant women–all with a benznidazole regimen. While there is a clear need to disaggregate data based on gender and other important demographic variables, many of the studies in this review did not differentiate when reporting outcomes, or did not report information on gender at all [44].

Understanding the landscape of the currently available data allows us to clearly identify gaps in research where scarce resources should be channelled. This was also supported by a landscape analysis of Chagas cardiomyopathy research activity in 2018, which similarly identified a trend of increased Chagas research over the investigated time period (1980–2016) and an overlapping geographic distribution of the research conducted as found in our review, with the majority of reports (82.9%) from Latin America and the Caribbean [43]. While the González-Alcaide et al. (2018) review observed an increase in international and particularly regional collaborations in recent years, with less than 1% of Chagas patients able to access treatment, the need for even greater efforts and resources to support capacity in research collaborations, such as the proposed IPD platform, is critical [45].

### Limitations

Only one reviewer was responsible for the initial title and abstract screening of the literature. There were also a number of full-text publications that could not be retrieved for assessment of eligibility that were conservatively excluded from analysis (n = 30). Furthermore, the review could only identify published studies, while valuable unpublished observational data that could provide additional insight into the disease remain very difficult, if not impossible, to find. The poor reporting observed in some publications had implications in terms of grading the quality of evidence that can generated from these studies (See S5 Table). Only 3 (2.8%) of the studies were deemed to be of high quality, 7 (6.4%) of moderate quality and the remaining 99 (90.8%) were deemed to be low or very low quality. Such poor reporting also limits our understanding of their design and the treatment outcomes assessed. This finding could be overcome in the future by the Chagas disease research community, policy makers and medicine regulatory agencies, with collaborative development of tools and resources that align with standards developed by the Clinical Data Interchange Standards Consortium (CDISC). This would allow a minimal common way of capturing, measuring and reporting Chagas disease research and, if adequately disseminated, should allow an improvement of data quality in prospective clinical research studies over time.

### Future research

Results from this systematic review demonstrate that the development of an IPD Chagas disease data platform for clinical research would enable optimisation of existing data and permit more in-depth analyses to strengthen evidence for treatment and diagnosis of Chagas disease and inform prospective data collection. In addition, risk factors associated with long-term progression of disease remains relatively under-studied. A repository with a harmonised data protocol to standardise datasets from clinical studies will thus provide an opportunity to explore such risk factors.

There are several challenges in setting up a data sharing platform including non-availability of data (e.g., electronic dataset non-existent, corrupted or lost) or reluctance to share data [18,46]. Developing a network with strict ethics, governance and security standards, which would enable equitable data sharing, reuse and analysis, therefore remains the key. Involvement of public institutions and governments of countries where Chagas disease is a public health issue will be crucial to ensuring the long-term sustainability of a Chagas data sharing platform, and in helping ensure research meets the most pressing public health demands. In partnership with DND*i*, engagement with the Chagas scientific research community will be essential to assemble data and maximise the re-use of data made available on a Chagas clinical research data platform. At the encouragement (or mandate) of research funders and publishers, the volume of data being shared for secondary reuse is growing [21,30–36]. However, researchers and research data from low- and middle-income countries are often under-represented in the data sharing landscape. In the context of infectious diseases, it is these communities, researchers, and institutions that are, for the most part, the primary data generators. A FAIR (Findable, Accessible, Interoperable, Reusable), equitable and sustainable data network for infectious diseases needs to represent all the evidence generated and would start with enhancing the searchability, discoverability and persistence of metadata from all relevant infectious disease data, not simply those studies for which data has been shared. In this space, focusing solely on access to data shared by industry, known researchers and well-established academic groups, or identifying relevant data from clinical trial registrations alone, is myopic and limits the potential for re-using and analysing the wealth of valuable data available globally.

Robust science requires review and consideration of the full evidence-base available on a given topic. As such, the findability of all relevant literature and datasets is a critical first step to addressing any research question. It is also a time-consuming step and one that is constantly duplicated by researchers prior to identifying and requesting relevant data. For this reason, and as a next phase to this systematic review, IDDO seeks to minimise this universal inefficiency by conducting regularly scheduled search updates and providing ongoing results to this project in a living systematic review (LSR) format [47,48]. These results (including reproducible search methodologies) will be made publicly available in a searchable graph database where researchers will have ready access to knowledge on all published literature and data generated globally. Similar LSRs are available for IDDO themes including the VL Surveyor, WWARN Malaria Clinical trial library and COVID-19 Clinical Trial LSR.

### Conclusions

This systematic review has demonstrated the breadth of Chagas clinical research data available from the assessment of trypanocidal treatments, to longitudinal observational studies and research assessing the many symptomatic interventions required for management of sequelae associated with this chronic disease. Notwithstanding the substantial heterogeneity in methodology and treatment regimens assessed in Chagas clinical studies over the past 20 years, this investigation has validated the technical value and potential for establishing an IPD platform as well as the need to do so. Rapid assembly of existing data on a FAIR, equitable and sustainable platform requires engagement and participation from the Chagas research community and will ensure the success of this initiative in generating new evidence and innovative resources that enable research-driven responses to the major challenges of Chagas disease.

## Supporting information

S1 Text PRISMA Checklist. (DOC)

S2 Text Search Strategy and Eligibility criteria. (DOCX)

S1 Data REDCap data dictionary and variable dictionary for data collection. (XLSX)

S2 Data Search Results. (XLSX)

S3 Data Source data supporting the systematic review of Chagas disease trypanocidal treatment studies. (XLSX)

S1 Table Diagnostic criteria used for patient enrolment by phase of the disease. (DOCX)

S2 Table Benznidazole dose variability by age group. (DOCX)

S3 Table Total number of parasitological assessments during study follow-up. (DOCX)

S4 Table Time point at which parasitological assessments were carried out during follow-up. (DOCX)

S5 Table Assessment of quality of a body of evidence using GRADE approach. (DOCX)
