# Supplementary material for: The Chagas disease study landscape: A systematic review of clinical and observational antiparasitic treatment studies to assess the potential for establishing an individual participant-level data platform
Source: PLoS Negl Trop Dis. 2021 Aug 16;15(8):e0009697. doi: 10.1371/journal.pntd.0009697 (PMC8428795; doi:10.1371/journal.pntd.0009697)

# **SUPPLEMENTARY 2_ SEARCH STRATEGY**

### SEARCH STRATEGIES BY ELECTRONIC BIBLIOGRAPHIC DATABASE

### Pubmed (Medline)

1. "trypanosoma cruzi"[Title/Abstract]
2. trypanosoma cruzi[MeSH Terms]
3. chagas[Title/Abstract]
4. chagas disease [MeSH Terms]
5. "american trypanosomiasis" [Title/Abstract]
6. american trypanosomiasis[MeSH Terms]
7. #1 OR #2 OR #3 OR #4 OR #5 OR #6
8. #7 Filters: Systematic Reviews
9. "editorial"[Publication Type]
10. "historical article"[Publication Type]
11. "letter"[Publication Type]
12. "review"[Publication Type]
13. #9 OR #10 OR #11 OR #12
14. #7 NOT #13
15. #14 OR #8
16. #15 AND ("1997"[Date - Publication] : "3000"[Date - Publication])
17. animals [mh] NOT humans [mh]
18. #16 NOT #17

*Update search additional terms: 19. limit 18 to yr="2017 -Current" (1501)*

**Lilacs** <http://lilacs.bvsalud.org/en/>

1. (tw:("Trypanosoma cruzi" OR "Chagas" OR "American trypanosomiasis"))

Filters applied:

Database – LILACS

Limits - Humans

*Update search additional terms: 2017*

*2018*

*2019*

**Cochrane Database of Systematic Reviews and Central Register of controlled trials**

1. "Trypanosoma cruzi" OR "Chagas" OR "American trypanosomiasis"

*Update Search additional terms: Limits: 01/01/2017 -31/12/2019*

### Embase

1. "chagas disease":ti,ab.
2. "trypanosoma cruzi".ti,ab.
3. american trypanosomiasis.ti,ab.
4. exp chagas disease/
5. Trypanosoma cruzi/
6. 1 or 2 or 3 or 4 or 5
7. limit 6 to (editorial or letter or "review")
8. 6 not 7
9. limit 6 to "reviews (maximizes specificity)
10. 8 or 9
11. limit 10 to human
12. limit 11 to yr="1997-Current"

*Update search edits to row 12: limit 11 to (human and yr="2017 - 2019") (1297)*

### ClinicalTrials.gov

1. "Trypanosoma cruzi" OR "Chagas" OR "American trypanosomiasis"

*Update search: Manually deleted references with pre-2017 dates.*

### WHO ICTRP

1. Trypanosoma cruzi OR Chagas OR American trypanosomiasis

*Update search: Manually limited to references added from 2017-2019.*

### RESULTS OF THE LITERATURE SEARCHES

| **Database** | **Original search results**  **7-11/09/2017** | **Update search results 23/08/19**  [2017 to date of search] |
| --- | --- | --- |
| Ovid Medline | 6,195 | 1501 |
| LILACS | 3,733 | 51 |
| Cochrane Database and CENTRAL | 178 | 62 |
| Ovid Embase | 6,209 | 1297 |
| Clinicaltrials.gov | 57 | 25 |
| WHO ICTRP | 70 | 19 |
| Total | 16,315 | 2955 |
| **Total after deduplication** | **10,319** | **2145** |

### Eligibility Criteria

#### PICOS Search String Strategy

| **PICOS** | **Search String Strategy** |
| --- | --- |
| Population | Disease terms including Chagas, Chagas disease trypanosoma cruzi; american trypanosomiasis were used. Where sensitive (Embase, PubMed, Lilacs databases only) additional filters were used to exclude animal studies. |
| Intervention | The search strategy was not restricted by intervention given the desire to also capture non-intervention studies |
| Comparator | The search strategy and eligibility for inclusion was not restricted by comparator given the desire to also capture non-intervention studies (i.e. longitudinal observational studies). |
| Outcomes | The search strategy and eligibility for inclusion was not restricted by treatment outcomes given the desire to compare and to understand the homogeneity/heterogeneity of all outcomes measured in Chagas disease. |
| Study Design | The search strategy was not restricted by study design given the desire to capture all possible study designs. Where sensitive (PubMed database online) non-primary research publication types were excluded "editorial"; "historical article"; "letter" |

#### PICOS Screening Strategy

| **PICOS Criteria** | **INCLUDED** | **EXCLUDED** |
| --- | --- | --- |
| **Population** | | |
|  | **INCLUDED POPULATION** | **“WRONG POPULATION”** |
|  | CHAGAS DISEASE   - enrolling human patients with a confirmed Chagas diagnosis - no limits were applied to participant age or Chagas disease phase | NOT HUMAN   - Animal Studies - pre-clinical studies   NOT CHAGAS   - no confirmed diagnosis of Chagas disease |
|  | **INCLUDED QUANTITATIVE ANALYSIS** | **OTHER** |
|  | - at least a subset of participants (n>6) was positively diagnosed for infection with Chagas, prior to receiving trypanocidal treatment - a post-treatment (>24hours) diagnostic test was performed on the same participants to assess outcome | - autopsy sample studies - transplant reactivation studies - Fewer than 6 Chagas patients enrolled |
| **Intervention** | | |
|  | **INCLUDED** |  |
|  | Any intervention and comparator was included at the level of full-text screening. |  |
|  | **INCLUDED QUANTITATIVE ANALYSIS** | **“EXCLUDED FURTHER ANALYSIS”** |
|  | Trypanocidal treatment where there is follow-up observation and potential for individual patient data | - Symptomatic intervention studies - Prospective observational cohorts and prognostic studies with no intervention |
| **Study Design** | | |
|  | **INCLUDED STUDY DESIGN** | **“WRONG STUDY DESIGN”** |
|  | INTERVENTIONAL AND PROSPECTIVE OBSERVATIONAL STUDIES   - - Clinical trials     - RCTs     - Quasi-RCTs - Prospective observational studies   - - Cohort studies/analysis     - Exposure 🡪 Outcome   - Prospective case series - Case-control   - - Outcome 🡪 Exposure | NON-PRIMARY RESEARCH STUDIES   - meta-analyses, reviews, textbook chapters, opinion papers and comments   DIAGNOSTIC STUDIES   - Diagnostic test accuracy (sensitivity/specificity etc) - Diagnostic prediction models   PROGNOSTIC PREDICTIVE STUDIES/MODELS   - Aim to predict future events - Model to inform therapeutic decision making - Retrospective cohort studies which designed to understand/look for prognostic markers   OTHER OBSERVATIONAL   - Retrospective studies - Case reports and retrospective case series - Epidemiologic studies   - Prevalence/Incidence   - Seroprevalence   - Risk of infection etc. - Cross-sectional   - disease and exposure status measured simultaneously in a given population   - Cross-sectional classification   - Clinical profile   - Largely prevalence - Snapshot, single point   SURVEILLANCE   - Vector transmission, risk of infection etc. - vector control, nets, vaccines or prophylaxis   THESIS |
|  |  | **“WRONG PUBLICATION DATE”** |
|  |  | - Article published prior to 1997 - Studies completed or concluding prior to January 1998 - Abstracts of conferences run before 1 January 2014 to be excluded, owing to the difficulty in establishing whether earlier abstracts were duplicate records of articles published within the considered time period. |
|  |  | **“EXCLUDED FURTHER ANALYSIS”** |
|  |  | - Ongoing studies - Abstracts for which no subsequent full-text is available |

Note: The eligibility for inclusion was not restricted by treatment outcomes given the desire to compare and to understand the homogeneity/heterogeneity of all outcomes measured in Chagas disease.

### PRISMA Diagram


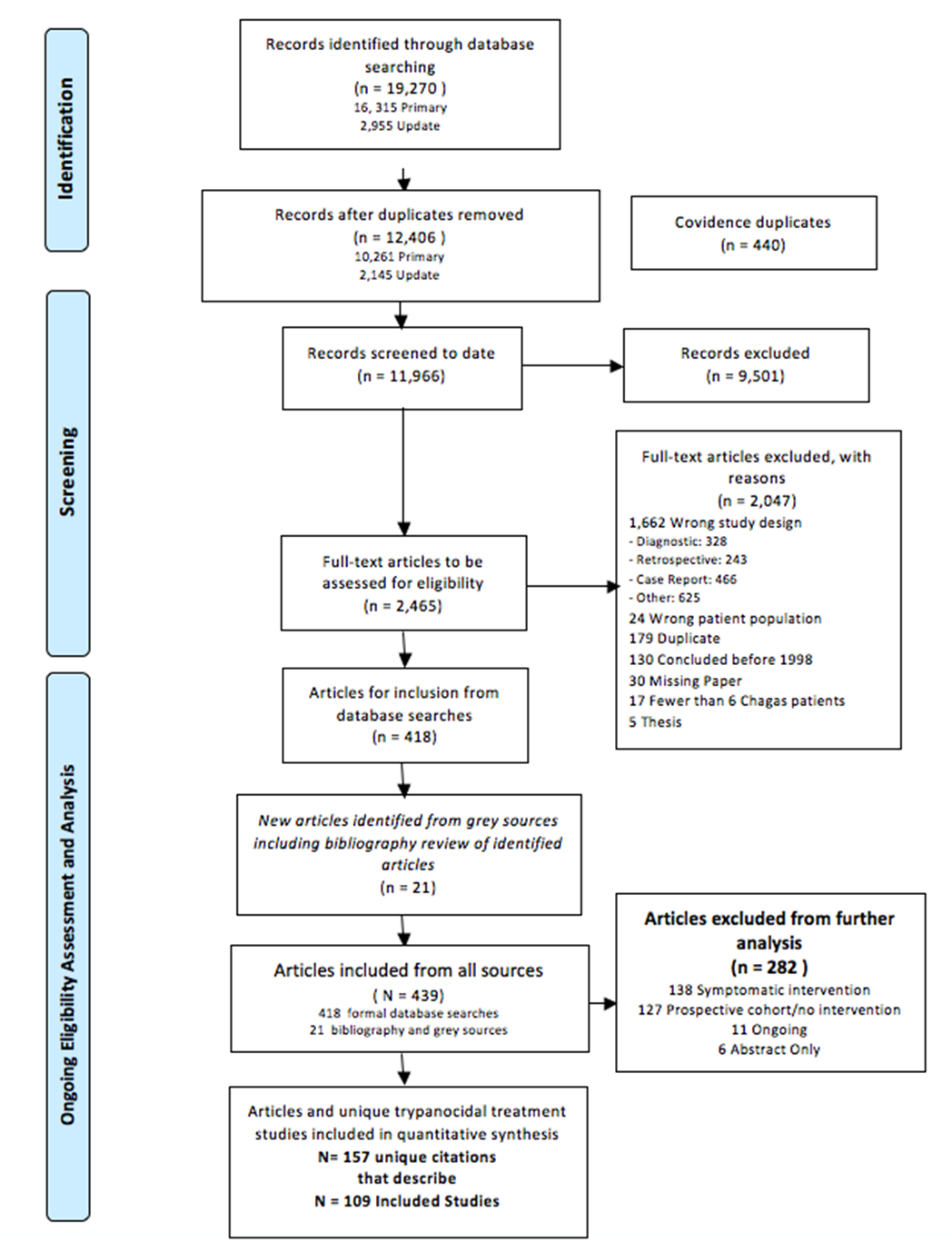

Supplement: S2 Text — (DOCX) [file pntd.0009697.s002.docx]
