# Supplementary material for: The Chagas disease study landscape: A systematic review of clinical and observational antiparasitic treatment studies to assess the potential for establishing an individual participant-level data platform
Source: PLoS Negl Trop Dis. 2021 Aug 16;15(8):e0009697. doi: 10.1371/journal.pntd.0009697 (PMC8428795; doi:10.1371/journal.pntd.0009697)
Supplement: S1 Table — (DOCX) [file pntd.0009697.s006.docx]

S1 Table: Diagnostic criteria used for patient enrolment by phase of the disease

|  | Acute | Chronic | Mixture | Unknown |
| --- | --- | --- | --- | --- |
| Number of studies | 6 | 86 | 8 | 9 |
| **Diagnostic method used** |  |  |  |  |
| Parasitological | 1 (16.7%) | 1 (1.2%) | 0 (0%) | 0 (0%) |
| PCR | 1 (16.7%) | 1 (1.2%) | 0 (0%) | 0 (0%) |
| Serological | 0 (0%) | 48 (55.8%) | 2 (25.0%) | 5 (55.6%) |
| Combination of serology/parasitology/PCR | 4 (66.7%) | 31 (36.0%) | 6 (75.0%) | 1 (11.1%) |
| Not specified | 0 (0%) | 5 (5.8%) | 0 (0%) | 3 (33.3%) |
| **Number of positive test**  **results required for enrolment** |  |  |  |  |
| 1 | 3 (50.0%) | 2 (2.3%) | 0 (0%) | 0 (0%) |
| 2 | 1 (16.7%) | 48 (55.8%) | 7 (87.5%) | 4 (44.4%) |
| 3 | 0 (0%) | 14 (16.3%) | 0 (0%) | 0 (0%) |
| 4 | 0 (0%) | 3 (3.5%) | 0 (0%) | 0 (0%) |
| >4 | 0 (0%) | 1 (1.2%) | 0 (0%) | 0 (0%) |
| Not specified | 2 (33.3%) | 18 (20.9%) | 1 (12.5%) | 5 (55.6%) |

Percentages are based on columns; PCR = polymerase chain reaction
