# Supplementary material for: The Chagas disease study landscape: A systematic review of clinical and observational antiparasitic treatment studies to assess the potential for establishing an individual participant-level data platform
Source: PLoS Negl Trop Dis. 2021 Aug 16;15(8):e0009697. doi: 10.1371/journal.pntd.0009697 (PMC8428795; doi:10.1371/journal.pntd.0009697)
Supplement: S2 Table — (DOCX) [file pntd.0009697.s007.docx]

S2 Table: Benznidazole dose variability by age group

| Benznidazole dosage | Number of study arms | Total treated patients |
| --- | --- | --- |
| Adults |  |  |
| 5 mg/kg/day for 30 to 60 days | 7 | 631 |
| 5 mg/kg/day for 60 days | 24 | 2299 |
| 5 mg/kg/day for 80 days | 1 | 1431 |
| 5 to 10 mg/kg/day for 60 days | 1 | 162 |
| >10 mg/kg for 60 days | 2 | 51 |
| Other | 1 | 6 |
| Unclear | 8 | 663 |
| Overall (adults) | 44 | 5,243 |
| Children including neonates |  |  |
| 5 mg/kg/day for 60 days | 2 | 118 |
| 5-10 mg/kg/day for 30 days | 2 | 121 |
| 5-10 mg/kg/day for 60 days | 5 | 145 |
| Unclear | 1 | 21 |
| Overall (children) | 10 | 405 |

Data from studies that used benznidazole in combination with another drug is not included in this table
