# Supplementary material for: The Chagas disease study landscape: A systematic review of clinical and observational antiparasitic treatment studies to assess the potential for establishing an individual participant-level data platform
Source: PLoS Negl Trop Dis. 2021 Aug 16;15(8):e0009697. doi: 10.1371/journal.pntd.0009697 (PMC8428795; doi:10.1371/journal.pntd.0009697)
Supplement: S3 Table — (DOCX) [file pntd.0009697.s008.docx]

S3 Table: Total number of parasitological assessments during study follow-up

| Number of parasitological  assessments | Number of studies | Percentage  (N=85 studies) |
| --- | --- | --- |
| 1 to ≤5 tests | 44 | 51.8% |
| 5 to ≤10 tests | 16 | 18.8% |
| 10 to ≤15 tests | 6 | 7.06% |
| >15 tests | 8 | 9.41% |
| Not specified | 11 | 12.9% |
