# Supplementary material for: The Chagas disease study landscape: A systematic review of clinical and observational antiparasitic treatment studies to assess the potential for establishing an individual participant-level data platform
Source: PLoS Negl Trop Dis. 2021 Aug 16;15(8):e0009697. doi: 10.1371/journal.pntd.0009697 (PMC8428795; doi:10.1371/journal.pntd.0009697)
Supplement: S4 Table — (DOCX) [file pntd.0009697.s009.docx]

S4 Table: Time point at which parasitological assessments were carried out during follow-up

| Parasitological  assessment | Median (days)  [Interquartile range] | Number of studies |
| --- | --- | --- |
| 1^st^ | 79 [30-180] | 74 |
| 2^nd^ | 180 [60-500] | 63 |
| 3^rd^ | 270 [180-545] | 53 |
| 4^th^ | 365 [218-730] | 42 |
| 5^th^ | 545 [240-1002] | 35 |
| 6^th^ | 655 [308-1095] | 30 |
| 7^th^ | 670 [360-1264] | 24 |
| 8^th^ | 820 [352-1372] | 20 |
| 9^th^ | 1095 [420-1620] | 17 |
| 10^th^ | 1275 [652-1812] | 15 |
| 11^th^ | 1308 [765-1958] | 14 |
| 12^th^ | 1368 [698-2190] | 12 |
| 13^th^ | 1132 [825-2144] | 10 |
| 14^th^ | 1050 [820-1458] | 8 |
| 15^th^ | 1110 [850-1549] | 8 |
