# Supplementary material for: The Chagas disease study landscape: A systematic review of clinical and observational antiparasitic treatment studies to assess the potential for establishing an individual participant-level data platform
Source: PLoS Negl Trop Dis. 2021 Aug 16;15(8):e0009697. doi: 10.1371/journal.pntd.0009697 (PMC8428795; doi:10.1371/journal.pntd.0009697)
Supplement: S5 Table — (DOCX) [file pntd.0009697.s010.docx]

S5 Table: Assessment of quality of a body of evidence using GRADE approach

| Study | Levels of quality of a body of evidence  (assessed using the GRADE approach) |
| --- | --- |
| Abramson 2013 | Low |
| Albareda 2015 | Low |
| Albareda 2018 | Low |
| Aldasoro 2015 | Low |
| Aldasoro 2018 | Low |
| Altcheh 2003 | Low |
| Altcheh 2005 | Low |
| Altcheh 2011 | Low |
| Altcheh2014 | Low |
| Altclas2005 | Low |
| Alvarez2016 | Low |
| Andrade 2013 | Low |
| Andrade 2016 | Low |
| Apt1998 | High |
| Bahamonde 2002 | Low |
| Balouz 2017 | Low |
| Barbosa 2016 | Low |
| Bertocchi 2013 | Low |
| Bianchi2015 | Low |
| Cardoso 2018 | Low |
| Chippaux2013 | Moderate |
| Coronado2006 | Low |
| Correia 2017 | Low |
| Crespillo-AndÃºjar 2018 | Low |
| Cutrullis2011 | Low |
| Da Silva Marques 2003 | Low |
| Das Neves Pinto2009 | Very Low |
| de Castro2006 | Low |
| Egui 2019 | Low |
| Fernandes2009 | Low |
| Fernandez-Villegas 2011 | Low |
| Fernandez-Villegas2016 | Low |
| Fernandez2016 | Very Low |
| Flevaud 2015 | Low |
| Flores-Chavez2006 | Very Low |
| Forsyth2016 | Low |
| Francisco-Gonzalez 2017 | Low |
| Fumado 2014 | Low |
| Garcia-Bournissen2015 | Low |
| Gorgolas 2013 | Low |
| Gray 2018 | Very Low |
| Jackson2010 | Low |
| Lacunza2006 | Very Low |
| Lacunza 2015 | Low |
| Laucella2009 | Moderate |
| Llaguno 2019 | Low |
| Martinez-Perez2016 | Low |
| Mateus 2017 | Low |
| Meira 2006 | Low |
| Molina2014; CHAGASAZOL | Low |
| Molina2015 | Low |
| Momper 2019 | Very low |
| MonjeRumi2013 | Low |
| Moreira 2015b | Low |
| Morillo2015; BENEFIT trial | Moderate |
| Morillo2017_2; STOP-CHAGAS | Moderate |
| Moroni 2019 | Very Low |
| Munoz 2006 | Low |
| Munoz2013 | Low |
| Munoz 2019 | Low |
| Murcia2016 | Low |
| Murcia2017 | Low |
| Niborski2016 | Low |
| Oliviera 2010 | Low |
| Ortiz 2019 | Low |
| Perez-Anton 2018 | Very Low |
| Perez-Ayala2011 | Low |
| Perez-Mazliah2013 | Very Low |
| Pinazo 2010 | Low |
| Pinazo 2013 | Low |
| Pinazo2016 | Low |
| Pontes 2010 | Very Low |
| Pupulin 2003 | Low |
| Rassi1999 | Very Low |
| Rassi 2000 | Low |
| Rassi 2002 | Low |
| Rassi2007 | Very Low |
| Ribeiro 2010 | Low |
| Robello 2019 | Low |
| Ruiz Lancheros 2018 | Low |
| Salvador2015 | Low |
| Sanchez 2016 | Low |
| Sanchez Negrette2008 | Very Low |
| Sartor2017 | Low |
| Sartori 2007 | Low |
| Schijman2003 | Low |
| Silveira2000 | Low |
| Simon 2019 | Low |
| Solari1998 | Very Low |
| Solari2001 | Low |
| SosaEstani1998 | Moderate |
| Sosa Estani 2004 | High |
| Soverow 2019 | Low |
| Soy2015; CINEBENZ | Low |
| Sperandio da Silva 2017 | Low |
| Streiger2004 | Low |
| Suarez 2005 | Low |
| Tornheim2013 | Moderate |
| Torrico2017 | High |
| Valente 2009 | Low |
| Vallejo2016 | Low |
| Velasquez 2019 | Low |
| Venegas 1997 | Low |
| Vera de Bilbao2004 | Low |
| Viotti2006 | Moderate |
| Viotti2011 | Low |
| Wendling 2011 | Low |
| Yun2009 | Low |
| Zulantay 1998 | Low |
